# Cumulative Sun Exposure and Melanoma in a Population-Based Case–Control Study: Does Sun Sensitivity Matter?

**DOI:** 10.3390/cancers14041008

**Published:** 2022-02-16

**Authors:** Leslie K. Dennis

**Affiliations:** 1 Department of Epidemiology and Biostatistics, Mel and Enid Zuckerman College of Public Health, University of Arizona, Tucson, AZ 85724, USA; ldennis@arizona.edu; 2 Department of Epidemiology, College of Public Health, University of Iowa, Iowa City, IA 52242, USA

**Keywords:** cumulative sun exposure, cutaneous melanoma, skin color, sun exposure, sun sensitivity, total sun exposure

## Abstract

**Simple Summary:**

Melanoma has been clearly shown to be related to sunburns and other types of intermittent sun exposure. It is less clear how cumulative sun exposure is related to melanoma. In this case–control study, the cumulative hours of sun exposure per day were examined between spring and fall each year over periods or decades of life to estimate lifetime hours of sun exposure. No associations were found before the age of 60. However, when stratified by fair skin color, we found little or no association with hours of sun exposure among fair-skinned individuals, but found an increased risk for higher hours of sun exposure among medium- or darker-skinned individuals for lifetime exposure.

**Abstract:**

Cutaneous melanoma (CM) has consistently been associated with intermittent sun exposure, while the association with chronic sun exposure is debated. The goal of this research was to examine the complex relationship between CM, sun sensitivity and sun exposure based on theoretical concepts of how these factors may be associated. Detailed sun exposure histories across life periods and various measures of sun sensitivity were collected in a population-based case–control study of melanoma in Iowa, USA. Participants were asked about their hours of sun exposure per day between March and October each year over periods or decades of life to estimate cumulative lifetime hours of sun exposure. Increased odds ratios (ORs) for CM were seen for most standard measures of sun sensitivity except for the tendency to sunburn. Minimal associations were seen with total hours of sun exposure early in life. However, an interaction was seen between fair skin color and lifetime hours of sun exposure, where the strongest associations with CM were seen among medium-skinned and dark-skinned participants. This suggests that cumulative sun exposure at high levels may increase CM among non-sun-sensitive individuals typically at lower risk of CM. Such a finding has implications for the prevention effort for melanoma regarding time in the sun among darker-skinned individuals.

## 1. Introduction

Skin cancer is caused by damage from ultraviolet radiation (UVR), a form of non-ionizing radiation emitted by the sun. The amount of UVR at a location is affected by cloud cover, altitude, altitude and time of year. Different forms of UVR are related to different types of skin cancer, leading to the Surgeon General’s Call to Action to Prevent Skin Cancer [1]. The highest worldwide rates of cutaneous melanoma (CM) are in Australia, which has high amounts of UVR. Patterns regarding distance from the equator (latitude) are less clear, due to the susceptibility of UVR damage by skin sensitivity to the sun, with higher melanoma rates in Scandinavia (19/100,000 in 2012) than in Mediterranean countries (11/100,000 in 2012), where many residents are less sun sensitive [2,3,4,5]. In the US, for 2013–2017, the rate of CM was 17.4 per 100,000 adjusted to the WHO world standard population [6]. CM has consistently been associated with intermittent sun exposure measures via sunburns or sunny vacations during childhood, adolescence, adulthood and over a lifetime [7,8,9]. This is complicated as sun-sensitive individuals (fair skin, red or blond hair color, tendency to sunburn on first exposure to the sun in spring/summer, and unable to tan) are at higher risk of melanoma and of sunburns [10]. For studies only reporting on ever vs. never having a sunburn during a specific time period, childhood has a stronger association with CM than other time periods, but receiving a higher number of sunburns at any time period is more important [11]. Chronic sun exposure has an unclear relationship to the development of CM due to conflicting results of studies which could imply effect modification by sun sensitivity [8,9,12,13,14]. One of the major issues when studying chronic sun exposure is that it can include a lot of different specific forms of sun exposure that are not comparable. Additionally, some forms of sun exposure can be chronic or intermittent depending on individual exposures and geographic locations (such as outdoor recreational or leisure activities that are seasonal in some places but year around in other places). Studies of high-occupation sun exposure typically show a reduced risk of melanoma [8,12], which may reflect self-selection for working outdoors by those who are not sun sensitive.

Early on, Armstrong [15] suggested that this relationship is complex as it relates to skin pigmentation, sun-seeking behavior and a closer proximity to the equator (ultraviolet radiation by geography). His theory suggests that sun-sensitive individuals’ risk of melanoma increases immediately with early sun exposure (which probably causes sunburns) then decreases (inverse U-shaped curve), unless they continue to be exposed to the sun over a longer period of time [15]. It is commonly thought that initial sun exposure in sun-sensitive individuals could create a sunburn or other damage and deter such individuals from further unprotected sun exposure. Armstrong [15] speculated that for those with a low pigment response to the sun (darker skin tone), their risk of melanoma only increases with cumulative sun exposure and those with an average pigmentary response are somewhere between the other two groups. If this is true, it would be tricky to model but could account for conflicting reports [8,9,12,13,14] due to a population’s mixture of individuals with different pigmentary responses that are not fully accounted for in many analyses. More recently, a couple of dual or divergent pathway hypotheses have been put forward, but interestingly such hypotheses began with a mathematical model looking at ambient UV radiation [7]. Green [16] proposed that the susceptibility of melanocytes to malignant alteration may vary by body location, explaining some differences related to intermittent sun exposure. Whiteman et al. [17] found genetic overexpression among those with an inability to tan, history of melanoma on the limbs or head but least frequent among those with heavy freckling or multiple nevi. They revised the dual-pathway hypothesis in 2003 to say that one pathway was associated with proliferation of melanocytes (nevi) and melanoma of the trunk and the other with chronic sun exposure and melanoma of the head and neck [18]. However, few studies have reported chronic sun exposure as a hours of sun exposure to be able to look at increases. The purpose of this study was to examine CM and hours of sun exposure over a lifetime using a case–control design among Iowa residents to look for patterns and to look for variations by sun sensitivity in order to see which hypothesis fits these data.

## 2. Materials and Methods

CM cases, with patients being at least 20 years old, able to speak English, residents of Iowa, and newly diagnosed (recruited within 9–12 months of diagnosis) with a microscopically confirmed CM diagnosed from January 2010 through to December 2012, were eligible. For cases, the State Health Registry of Iowa (Iowa *Surveillance, Epidemiology, and End Results* cancer registry) used semi-rapid reporting methods to obtain as many cases as possible within 6–12 months after diagnosis. CM cases were contacted via letter by the State Health Registry of Iowa to be asked if they were interested in participating in this study. This was followed by a phone call to recruit subjects for a computer-assisted telephone interview.

Population-based controls were randomly selected and frequency matched on age and sex from the Iowa Voter Registration file. In Iowa, the voter registration file was found to be population-based due to the high level of registration. The Iowa Driver’s License records, a population-based source in Iowa that has previously been used for research, compared to the Iowa Voter Registration file had 96.3% of the overall number of individuals listed in the driver’s license records appearing in the Iowa Voter Registration file (100.2% for females, suggesting more females are registered to vote in Iowa than those who have driver’s licenses, and 92.3% for males). The number of residents listed in the Iowa Voter Registration file was also similar to Census data by age and sex.

**Figure 1:**
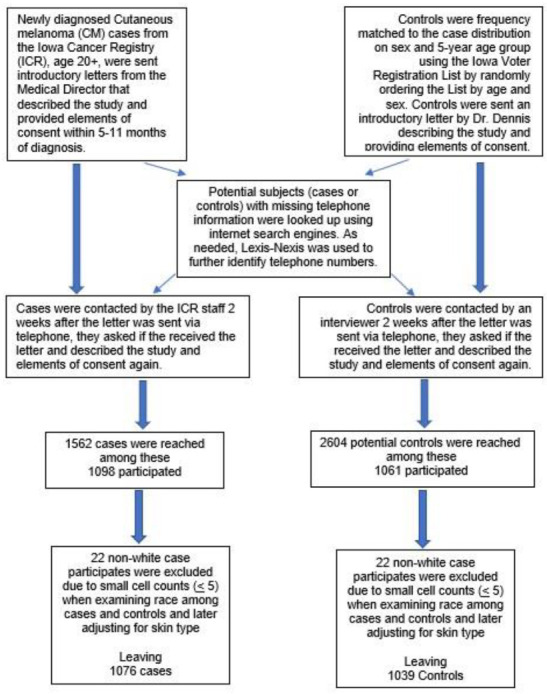
Recruitment of subjects.

All subjects were recruited to participate in a computer-assisted telephone interview (Figure 1). The interviewers asked about hours per day spent in the sun from both work and leisure time, on average from March to October for various life periods including before high school (childhood), during high school (adolescence), ages 18–21, ages 22–29, and then decades of life (ages 30–39, …, 70–79, and age 80+). The months from March to October were chosen based on average temperatures that would lead to clothing habits that would exposure skin to sunlight (t-shirts and shorts). Based on the length of sunlight in the US and in Iowa, a maximum of 15 h of sunlight occurred. To estimate cumulative sun exposure during each of these time periods, the years spent in each time period based on age were multiplied by 245 days (between March and October) times the reported hours spent outside per day. This allowed for the summation of cumulative hours spent in the sun across 20 years (ages 0–21, 22–29, 40–59, and 60+) and over a lifetime. These were than categorized into quartiles based on all White subjects and rounding to the nearest 50. These analyses were restricted to the 98% who reported being White, similar to other studies of melanoma, but analyses including non-Whites had similar results.

Sun sensitivity is an important potential confounder measured in multiple ways. Subjects were asked about their sun sensitivity including tendency to sunburn at first exposure in the spring or summer with no protection (severe, moderate, mild or no burn), inability to tan after repeated and prolonged sun exposure (no tan, mild, moderate or deep tan), self-reported skin type, skin color of the upper inner arm (fair, medium or dark), hair color (blond, strawberry blond, red, auburn, brown or black), if their hair was light, medium or dark, and eye color. Subjects who reported hazel eye color were informed that Webster’s Dictionary defines hazel as brown and asked what they meant by hazel to refine their eye color; then, eye color was categorized into blue or green compared to brown eye color. For hair color, blond and strawberry blond were combined, as were red and auburn. The University of Iowa IRB approved the recruiting and interviewing all subjects. Mid-recruitment, the University of Arizona approved recruitment of controls in Iowa to be interviewed for telephone by staff in Arizona.

Unconditional logistic regression analyses were used to estimate odds ratios (ORs) for the risk of CM by exposure factors and 95% confidence intervals (CI). Effect modification was examined by interaction terms with *p*-values < 0.05 for sex and sun sensitivity. Sun exposure was examined in quartiles. Confounding was examined as changes in the OR of >10%. Age was not adjusted for since the measures of cumulative hours of sun exposure were based on age, making it inappropriate then to adjust for age as it would be adjusting for part of our main exposure of interest. Based on univariate associations with CM, inability to tan (no, mild or moderate tanning vs. deep tanning), fair skin color (compared to medium or dark), red or blond hair (compared to brown or black) and light eye color (blue or green vs. brown) were examined as potential confounders along with sex.

## 3. Results

### 3.1. Population Characteristics and Sun Sensitivity

Table 1 presents sun sensitivity by CM. Sun-sensitive individuals (fair skin color, self-reported skin type of always burning and never tanning, inability to tan, blue or green eye color and red or blond hair) had higher ORs for CM. When medium and dark skin are combined, the OR for fair skin is 1.69 (95% CI: 1.39–2.05) compared to medium or dark-skin individuals. In this population, CM was not associated with the tendency to sunburn. CM increased with age and was higher in males.

### 3.2. Hours of Sun Exposure

Looking only at specific life periods (childhood, adolescence, ages 18–21, 22–29, and then decades), associations with melanoma were seen for 8+ hours/day spent in the sun (March through October) for ages 18–21 and 60–69, with suggested associations for ages 50–59 and 70–79 and with 5–7 h/day for ages 80+ (data not shown). To better examine associations with hours of sun exposure, we looked at cumulative hours spent in the sun across 20-year periods (ages 0–21, 22–29, 40–59, and 60+) and over a lifetime. Table 2 presents reported cumulative hours of sun exposure for ages 0–21, 22–29, and 40–59, controlled for sun sensitivity. For these hours of sun exposure early in life, few or no associations were seen. There was a 59% increase in the OR for the highest quartile for hours of sun exposure (22,050–93,100 h) for ages 40–59, but not for other quartiles.

The association of CM with hours of sun exposure for ages 60+ and lifetime interacted with skin color. Table 3 reports these associations stratified by skin color (fair vs. medium/dark skin). For ages 60+, fair-skinned individuals had an increasing risk with increasing hours of sun exposure, but the risk was even higher in medium-/dark-skinned participants. For lifetime sun exposure, the risk of CM was in medium-/dark-skinned participants. Lifetime sun exposure also showed an interaction with sex, showing that the association was among males. For lifetime sun exposure stratified by either skin color or sex, the other factor no longer showed an interaction. When stratifying by both skin color and sex, the strongest association was among medium-dark-skinned males with an OR = 2.86 (95% CI of 1.08–7.54) for the third quartile and an OR = 5.31 (95% CI of 2.10–13.4) for the fourth quartile.

Sensitivity analyses were run including non-Whites, showing similar results. While we had no power (wide CIs) to look at the 46 non-whites separately, the highest quartile for lifetime sun exposure had an OR = 3.5 for non-Whites.

## 4. Discussion

For this Iowa population that self-reports as mostly fair skinned (73%), we found an effect modification by quartiles of lifetime hours in the sun by skin color with little or no association among those self-classifying as fair skinned (OR = 1.4 for highest quartile), but increased ORs of CM with increased hours of sun exposure (for the third and fourth quartiles) among those with medium or dark skin. These total lifetime hours of sun exposure include both occupational sun exposure and non-occupational sun exposure. Such trends support Armstrong’s [15] speculated association of a CM increase with enough sun exposure among non-sun-sensitive people. This also supports the divergent pathway hypothesis by Whitman et al., [18] of chronic sun exposure causing CM along with a pathway associated with the proliferation of nevi. Therefore, considering sun sensitivity as an effect modifier or at least as a confounder in melanoma research is crucial. Those with a high pigmentary response, fair-skinned individuals, are at higher risk of CM but not due to many hours of sun exposure. For those with medium-dark skin, a low pigmentary response to the sun, the risk of CM increases only with enough cumulative hours of sun exposure. Other aspects of our results are consistent with prior studies that have shown CM risk to increase with sun sensitivity factors including fair skin color, inability to tan, and natural red or blond hair color, along with age and male sex. Although this population is predominantly fair skinned, only 6% reported experiencing a severe or painful sunburn when first exposed to the sun in the summer, 8% with skin type of always burning and never tanning with prolonged and repeated sun exposure, and 11% with red or auburn hair natural hair color at age 20.

Studies on cumulative or total hours of sun exposure have been conflicting. An increased risk of CM was seen in a Mediterranean study on nonfamilial cutaneous malignant melanoma with increasing hours of total sun exposure adjusted for age, skin color and skin type [19]. Holman et al. [20] looked at the mean annual hours of bright sunlight in Australia, finding a significant trend of ORs in CM risk with more hours among native-born Australians (so longer exposure) and a reduction among migrants; however, the adjustment for sun sensitivity was unclear. Another Australian study found increased CM with increasing tertiles of lifetime sun exposure only when inversely weighted by sunscreen use [21]. A study of a Southern European population saw an increased risk of melanoma for increasing weeks per year of sun exposure [22]. Another study of longshoreman in Italy reported they were at increased risk of CM, thought to be due to chronic occupational sun exposure [23]. In Spain, a study found that > 20 years of occupational sun exposure was related to increased CM [24]. A study in Germany [25] looked at a variety of recreational activities, finding some to increase CM risk and others to decrease it, but they did not report on the hours of sun exposure. White et al. [26] found a significant reduced risk of CM with higher hours of sun exposure at ages 2–10 and 11–20 among the deepest tanners, but did not measure lifetime or total sun exposure as reported here and in other studies. A New York study also saw a protective association with increasing average annual hours of sun exposure among males for 2101+ average annual hours/year, and found when stratifying by sex and complexion for total hours of sun exposure the protection was only among males with a light complexion [27]. This is not inconsistent with our finding since the average hours per year are not directly comparable with cumulative lifetime hours of sun exposure. Several other studies reported no association with CM and lifetime sun exposure [28,29,30,31,32]. However, some of the studies with no associations had small numbers and one did not report the actual hours of exposure for comparison. The HELIOS project [29] had a suggested U-shaped curve with the highest risk for > 22,000 lifetime hours of sun exposure. One study reporting lifetime hours of sun exposure had only 37+ for the highest tertile [30] and another only reported average hours/day so did not estimate a cumulative lifetime hours of sun exposure [32].

Chronic sun exposure has been measured as occupational exposure, in addition to total hours of sun exposure. Part of the conflict in prior studies is based on some of these distinctions, which measure different kinds of exposure. For occupations with high sun exposure, there is likely to be self-selection by sun sensitivity. When examining occupational sun exposure, a study in western Canada found a suggestive inverse U-shaped association with estimated occupational hours of sun exposure in the summer [33]. Similarly, in the Agricultural Health cohort (Iowa and North Carolina) among farmers, an inverse U-shaped curve was seen for hours per day in the sun during the growing season at enrollment with later development of melanoma, with a significant protective association for more than 10 h per day [34]. An increased risk of CM was seen for occupational exposure in a Mediterranean study, adjusted for age, skin color and skin type [19]. However, several other studies found no clear association with occupational sun exposure [31,35,36,37,38]. Loria and Matos [35] only looked at any hours of occupation exposure (21–9230 h), which might account for the lack of an association. Additionally, a study in the UK found a non-significant U-shaped curve, rather than an inverse U-shaped curve, for occupational sun exposure with the highest risk for 50,000+ hours outdoors [38]. A study in the Netherlands reported a reduced risk of CM among subjects reporting that they ever had an outdoor occupation [39], while another study suggested a reduction for lifetime occupational sun exposure at 50% or greater in a US study [26]. This dichotomous categorization provides no information of cumulative occupational hours of sun exposure. When comparing occupations of mostly indoors to most outdoors, there is likely to be some self-selection for fair-skin individuals who easily burn to not have most outdoor occupations.

A recent meta-analysis looked at UVR in “skin of color” and CM risk. Their definition of people of skin of color included all races/ethnicities except for non-Hispanic White individuals, Fitzpatrick skin type (IV-VI), or tanning ability (rarely or never burns) [40,41]. Of the 13 studies summarized, seven were ecological studies, and therefore the exposure and outcome were measured across populations, not within individuals, so could suffer from the ecological fallacy that those with the exposure did not have the outcome. Among the studies, they found five Asian populations, one Hispanic population and seven mixed racial/ethnic populations [41], with only two studies reporting associations (one for Hispanic males and another for Black males). Measures of UVR exposure reviewed were also heterogenous, including latitude, UV flux, UV index, UV irradiance, photochemotherapy and narrow-band UV-B phototherapy. The additional exclusion of non-English studies could have added other bias [41]. The studies summarized did not include any of the above-mentioned studies of hours of sun exposure and CM. While looking at CM in darker-skinned populations is important, it might be more prudent to summarize across analytic studies based on the Fitzpatrick skin type [40], but few studies have stratified by similar UVR exposure measures by skin-type. Additional studies in other populations are needed to further assess CM risk and cumulative hours of sun exposure over a lifetime in non-fair skinned individuals and populations. A prior meta-analysis of CM among populations of Spanish descent only identified seven studies, with none reporting measures of cumulative sun exposure so only ever sunburned, fair skin color and lighter skin type could be pooled with similar findings to non-Hispanic Whites [42]. Large studies on Hispanics, whom typically have a wide range of Fitzpatrick skin types, would significantly add to our knowledge.

If the increase seen here and in a few other studies for lifetime hours of sun exposure and CM is real, then cancer prevention efforts need to be carefully considered for long-term effects of sun exposure in non-fair skinned individuals and populations. Current skin cancer prevention efforts in Australia (with the highest rates of skin cancer) and the United States do address the length of time your skin is exposed as a concern when protecting yourself from harmful effect of the sun. However, the findings of this study suggest we need to continue efforts among tanners and dark-skinned individuals who spend a large amount of time in the sun.

A strength of our study is that it is moderate to large size population-based case–control study. Cases were recruited via semi-rapid reporting within about 9 months of diagnosis. Furthermore, sun sensitivity was asked in a variety of ways to best capture it. Participants had a wide range of sun exposure hours over their lifetime between spring and fall in which to gauge differences. Limitations include reporting bias suffered by all study designs, which includes some inaccuracy in recalling past exposures. Additionally, sunscreen use and clothing habits were not asked at all life periods due to concern about recall beyond the past five years, so could not be modeled within the cumulative sun exposure hours.

## 5. Conclusions

Our findings suggest that CM is associated with an increased OR for increasing hours of lifetime sun exposure in non-sun-sensitive individuals, so sun sensitivity does matter. The increases were highest among medium-/dark-skinned males. This suggests that cumulative sun exposure at high levels may increase CM among non-sun-sensitive individuals typically at lower risk of CM. More studies of CM among darker-skinned population are needed.

## Figures and Tables

**Table 1:** Host factors and sun sensitivity measures among 1076 melanoma cases and 1039 population-based controls in Iowa.

Host Factors	Cases ^1^	%	Controls ^1^	%	OR ^2^	95% CI	
**Age at interview**							
20–39	145	13.5	167	16.1	1.01	0.76	1.34
40–49	144	13.4	162	15.6	1.03	0.77	1.37
50–59	223	20.7	258	24.8	ref		
60–69	253	23.5	268	25.8	1.09	0.85	1.40
70–79	193	17.9	137	13.2	1.63	1.23	2.16
80+	118	11.0	47	4.5	2.91	1.98	4.26
**Sex**							
Female	523	48.6	567	54.6	ref		
Male	553	51.4	472	45.4	1.27	1.07	1.51
**Upper inner arm skin color**							
Fair	844	78.5	710	68.4	1.69	1.39	2.05
Medium/Dark	231	21.5	328	31.6	ref		
**Self-reported skin-type**							
Always burn/never tan	101	9.3	60	5.9	2.44	1.71	3.47
Usually burn, tan diff	222	20.4	167	16.0	1.92	1.49	2.48
Some burn, then tan	473	44.3	409	39.3	1.67	1.37	2.05
Rarely burn/tan easy	275	26.0	398	38.8	ref		
**Tendency to sunburn**							
A severe and painful sunburn	72	6.8	58	5.7	1.24	0.84	1.82
A moderate sunburn	265	24.9	231	22.6	1.14	0.89	1.46
A mild sunburn	470	44.1	478	46.7	0.98	0.79	1.21
No sunburn	258	24.2	257	25.1	ref		
**Ability to tan**							
Deeply tanned	161	15.0	161	21.0	ref		
Moderately tanned	448	41.7	448	46.5	1.26	0.99	1.60
Mildly tanned	347	32.3	347	26.4	1.72	1.33	2.23
Have no tan	118	11.0	118	6.1	2.54	1.76	3.66
**Eye color**							
Blue	538	50.1	438	42.4	1.61	1.30	1.99
Green	310	28.8	298	28.9	1.36	1.08	1.72
Brown or Black	227	21.1	297	28.8	ref		
**Natural hair color at age 20**							
Red	154	14.3	87	8.4	2.13	1.60	2.84
Blond	336	31.3	246	23.7	1.65	1.35	2.01
Brown or Black	585	54.4	705	67.9	ref		
**Hair shade**							
Light	345	32.1	254	24.5	1.98	1.57	2.50
Medium	497	46.3	444	42.9	1.63	1.32	2.01
Dark	232	21.6	338	32.6	ref		

CI = Confidence interval; OR = odds ratio; ref = reference group for odds ratios. ^1^ Numbers may not add to total due to missing values that are not included in percentages or ORs. ^2^ Crude Odds ratio as host factors are not typically adjusted.

**Table 2:** Hours of sun exposure between March and October over 20-year time periods and lifetime among 1076 melanoma cases and 1039 population-based controls in Iowa ^1^.

Sun Exposure	Cases	%	Controls	%	OR ^2^	95% CI	
**Hours of sun ages 0–21**							
0–15,200 h	251	23.3	278	26.8	ref		
15,201–23,500 h	268	24.9	244	23.5	1.22	0.96	1.56
23,500–33,800 h	284	26.4	255	24.5	1.28	1.00	1.63
33,801–78,900 h	273	25.4	262	25.2	1.23	0.96	1.57
Total	1076		1039				
			Trend OR ^3^		1.22	*p* = 0.097	
**Hours of sun ages 22–39**							
0–7350 h	255	23.9	273	26.7	ref		
7351–13,200 h	256	24.0	238	23.3	1.19	0.93	1.52
13,201–22,050 h	296	27.7	269	26.4	1.25	0.98	1.59
22,051–82,350 h	262	24.5	241	23.6	1.27	0.99	1.62
Total	1069		1021				
			Trend OR ^3^		1.26	*p* = 0.056	
**Hours of sun ages 40–59**							
0–5150 h	223	24.1	236	27.2	ref		
5151–11,750 h	216	23.3	216	24.9	1.11	0.85	1.45
11,751–22,050 h	233	25.2	224	25.8	1.22	0.94	1.59
22,050–93,100 h	254	27.4	191	22.0	1.59	1.22	2.09
Total	926		867				
			Trend OR ^3^		1.56	*p* = 0.0006	

CI= Confidence interval; h = hours; OR = odds ratio. ^1^ Numbers may not add to total due to missing values that are not included in percentages or ORs; for older age categories, missing values also represent individuals not in that age category and thus who cannot contribute to those categories. ^2^ Odds ratio adjusted for self-reported skin-type (always burn/never tan, usually burn, tan diff, some burn, then tan, vs. rarely burn/tan easy). ^3^ Trend odds ratio comparing the reference group to the highest category.

**Table 3:** Lifetime and age 60+ h of sun exposure between March and October among 1076 melanoma cases and 1039 population-based controls in Iowa stratified by self-reported skin color ^1^.

Sun Exposure	Fair Skin					Medium and Dark Skin				
	Cases	Controls	OR ^2^	95% CI		Cases	Controls	OR ^2^	95% CI	
**Hours of sun ages 60+**										
0–1950 h	83	86	ref			26	45	ref		
1951–5900 h	114	70	1.73	1.12	2.67	24	57	0.81	0.41	1.62
5901–14,200 h	103	76	1.59	1.03	2.47	36	31	1.94	0.97	3.89
14,201–79,400 h	120	59	2.72	1.72	4.30	52	21	4.81	2.34	9.87
Total	420	291				138	154			
	Trend OR ^3^		2.39	*p* < 0.0001		Trend OR ^3^		5.50	*p* < 0.0001	
**Lifetime Hours**										
0–31,500 h	211	194	ref			28	85	ref		
31,501–60,200 h	288	264	1.03	0.80	1.34	79	117	2.09	1.24	3.52
60,201–79,000 h	126	94	1.31	0.94	1.83	48	41	3.71	2.03	6.79
79,001–249,000 h	219	158	1.44	1.08	1.92	76	85	2.81	1.65	4.81
Total	844	710				231	328			
	Trend OR ^3^		1.49	*p* = 0.005		Trend OR ^3^		2.52	*p* = 0.001	
	**Females**					**Males**				
**Lifetime Hours**										
0–31,500 h	179	197	ref			60	82	ref		
31,501–60,200 h	216	234	1.07	0.81	1.41	151	148	1.41	0.94	2.12
60,201–79,000 h	69	65	1.24	0.83	1.85	106	70	2.23	1.41	3.51
79,001–249,000 h	59	71	0.99	0.66	1.50	236	172	2.08	1.40	3.09
Total	523	567				553	472			
	Trend OR ^3^		1.08	*p* = 0.681		Trend OR ^3^		2.01	*p* < 0.0001	

CI = Confidence interval; h = hours; OR = odds ratio; ref = reference group for odds ratios. ^1^ Numbers may not add to total due to missing values that are not included in the ORs; for older age categories missing values also represent individuals not in that age category and thus who cannot contribute to those categories and one subject missing skin color. ^2^ Odds ratios are stratified by skin color and adjusted for self-reported skin-type. ^3^ Trend odds ratio comparing the reference group to the highest category.

## Data Availability

Data available on request due to restrictions/confidentiality issues.
